# Mesenchymal Stem Cells Isolated from Paediatric Paravertebral Adipose Tissue Show Strong Osteogenic Potential

**DOI:** 10.3390/biomedicines10020378

**Published:** 2022-02-04

**Authors:** Jan Rožanc, Lidija Gradišnik, Tomaž Velnar, Minja Gregorič, Marko Milojević, Boštjan Vihar, Boris Gole, Uroš Maver

**Affiliations:** 1 Institute of Biomedical Sciences, Faculty of Medicine, University of Maribor, Taborska Ulica 8, 2000 Maribor, Slovenia; lidija.gradisnik@um.si (L.G.); marko.milojevic1@um.si (M.M.); bostjan.vihar@um.si (B.V.); 2 Department of Neurosurgery, University Medical Centre Ljubljana, Zaloska 7, 1000 Ljubljana, Slovenia; tomaz.velnar@um.si; 3 Department of Plastic and Reconstructive Surgery, University Medical Centre Maribor, Ljubljanska Ulica 5, 2000 Maribor, Slovenia; minja.gregoric@ukc-mb.si; 4 Center for Human Molecular Genetics and Pharmacogenomics, Faculty of Medicine, University of Maribor, Taborska Ulica 8, 2000 Maribor, Slovenia; boris.gole@um.si; 5 Department of Pharmacology, Faculty of Medicine, University of Maribor, Taborska Ulica 8, 2000 Maribor, Slovenia

**Keywords:** mesenchymal stem cells, paediatric surgery, paravertebral adipose tissue, regenerative medicine, stem marker expression, osteogenic potential, differentiation

## Abstract

Mesenchymal stem cells (MSCs) represent the basis of novel clinical concepts in cellular therapy and tissue regeneration. Therefore, the isolation of MSCs from various tissues has become an important endeavour for stem cell biobanking and the development of regenerative therapies. Paravertebral adipose tissue is readily exposed during spinal procedures in children and could be a viable source of stem cells for therapeutic applications. Here, we describe the first case of MSCs isolated from paravertebral adipose tissue (PV-ADMSCs), obtained during a routine spinal surgery on a child. Using quantitative real-time PCR and flow cytometry, we show that PV-ADMSCs have different levels of stem marker expression compared to the MSCs from other sources while having the highest proliferation rate. Furthermore, we evaluate the multipotency of PV-ADMSCs by the three-lineage (adipogenic, osteogenic and chondrogenic) differentiation and compare it to the multipotency of MSCs from other sources. It was found that the PV-ADMSCs have a strong osteogenic potential in particular. Taken together, our data indicate that PV-ADMSCs meet the criteria for successful cell therapy, defined by the International Society for Cellular Therapy (ISCT), and thus, could provide a source of MSCs that is relatively easy to isolate and expand in culture. Due to their strong osteogenic potential, these cells provide a promising basis, especially for orthopaedic applications.

## 1. Introduction

The isolation of mesenchymal stem cells (MSCs) from various tissue sources has become widely important in the rapidly developing field of regenerative therapies and tissue engineering for their broad clinical potential. MSCs are particularly interesting due to their intrinsic differentiation potential that is not found in other cells [1]. They are easily grown in culture dishes, produce an abundance of beneficial growth factors and cytokines and can differentiate into mesodermal tissue lineages such as cartilage, bone, muscle and adipose cells. [2].

The therapeutic potential of MSCs has been extensively studied over the past decade. However, its full clinical implementation has not yet been realised. Extensive research on MSCs has been conducted for regenerative therapies in orthopaedics, including repairing inherited, traumatic or degenerative injuries/changes of the bone, joints and cartilage tissue [3]. Other potential clinical applications include plastic surgery for muscle and adipose tissue reconstruction, e.g., after mastectomy or burns [4], and the tissue-healing properties of MSCs for locally acting inflammatory/immune-mediated tissue damage [5].

To date, MSCs have been isolated from many sources. MSCs from adults have been isolated from bone marrow, skeletal muscle, dental pulp, peripheral blood and adipose tissue. MSCs from neonatal sources were mostly isolated from sources like the umbilical cord, synovial membrane, placenta and amnion, to name a few [6]. Although bone marrow-derived MSCs are most commonly used in clinical trials [7], MSCs derived from neonatal and paediatric tissues can exhibit even more remarkable biological properties, such as high proliferative capacity, high differentiation potential and longevity [8]. Moreover, paediatric MSCs have shown potent immunomodulatory effects on the adaptive immune response [9] and could potentially even be used to combat viruses such as SARS-CoV-2 [10].

Adipose-derived mesenchymal stem cells (ADMSCs) have been most widely used for clinical applications in recent years because of their advantages in harvesting, isolation and expansion [11]. ADMSCs can be obtained through surgical procedures and direct excision or liposuction of the trunk and extremities, with either enzymatic separation of the cells or collection and processing of the stromal vascular fraction (SVF) to obtain the desired cells. Several ongoing phase 2 and 3 clinical trials are currently investigating the use of autologous ADMSCs in osteoarthritis (NCT03869229), spinal cord injury (NCT04520373), tendon injury (NCT02298023), Crohn’s disease (NCT01541579), and COVID-19-related acute respiratory distress (NCT04905836), to name a few.

Paravertebral adipose tissue (Supplementary Figure S1) is commonly encountered during neurosurgical spinal operations and it is partially removed when exposing the spinal bone, especially the vertebral laminas. The paravertebral adipose tissue is normally located deep under the paravertebral muscles close to the laminae. Its quantity varies among patients. In young children, the tissue is quite abundant and then gradually disappears during growth and ageing. In the elderly, paravertebral adipose tissue accumulates again due to ectopic fat infiltration into the lumbar paravertebral muscles. Its accumulation can also be observed in some pathological conditions with paravertebral muscle atrophy and degeneration [12,13]. In paediatric neurosurgery, treatment of tethered cord syndrome requires exposure of the spinal laminae before entering the spinal canal, opening the dura, and transecting the *filum terminale* that connects the spinal cord to the sacral bone [14,15]. During this elective spinal surgery, samples of paravertebral adipose tissue can be collected and used for cell isolation without additional risk to the patient. 

Here, we describe the first case of ADMSCs isolated from paravertebral adipose tissue in a child during tethered cord syndrome treatment and compare it with ADMSCs from other sources. We present an isolation procedure to obtain cells with high regenerative and differentiation properties that could potentially be included in the routine collection of stem cells for research, biobanking or therapeutic applications.

## 2. Materials and Methods

### 2.1. ADMSC Isolation and Culturing

#### 2.1.1. PV-ADMSC

The sample of paravertebral adipose tissue was obtained during an already planned elective spinal surgery in a 3-month-old child with tethered cord syndrome. Permission to use human tissue was obtained from the Committee of Medical Ethics of the University Clinical Centre Maribor, as was the patient’s written informed consent (UKC-MB-KME-120/13). Under sterile conditions, the adipose tissue fragments, approximately 1 cm^2^ in size, were transferred to phosphate-buffered saline (PBS) and transported to the laboratory. The tissue fragments were transferred from the transport centrifuge tubes to Petri dishes with a diameter of 3.5 cm and washed with sterile PBS. The tissue was cut into smaller fragments and incubated with 0.25 wt.% trypsin/EDTA (Merck KGaA, Darmstadt, Germany) for 30 min at 37 °C and in 5 wt.% CO_2_. Advanced DMEM (Thermo Fisher Scientific, Manassas, VA, USA) containing 100 IU/mL penicillin, 0.1 mg/mL streptomycin, 2 mM L-glutamine (all Merck KGaA, Darmstadt, Germany) and 5 wt.% heat-inactivated FBS (Thermo Fisher Scientific, MA, USA) was added. The suspension was centrifuged at 200× *g* for 5 min. The sediment was resuspended and transferred to T25 flasks. The growth medium was changed every three days. After 14 days of incubation, a monolayer of cells formed.

#### 2.1.2. LA-ADMSC

50–100 mL tumescent lipoaspirate samples were obtained during already planned breast reconstruction procedures in adult females. Written informed consent was obtained from the donors, and the study was approved by the National Medical Ethics Committee (approval 0120-349/2018/5).

Within 30 min, lipoaspirate samples were transferred to the cell culture laboratory and processed as previously described [16]. Briefly, samples were washed in Dulbecco’s-PBS to remove the blood, followed by digestion with collagenase Ia (Sigma-Aldrich, MI, USA). Samples were then centrifuged, and the upper fat layer and supernatant were discarded. The remaining pelleted stromal vascular fractions (SVFs) containing the MSCs were resuspended in DMEM/F12 GlutaMAX, supplemented with 10 wt.% FBS and 1 wt.% Pen/Strep (all Gibco, MA, USA) and filtered through a 70 µm cell strainer to remove undigested tissue remnants. The obtained cell suspensions were plated on cell culture flasks and subsequently cultured in supplemented DMEM/F12 GlutaMAX at 37 °C, 5 wt. % CO_2_, 90% RH. After 1 day, the culture medium was changed to remove the non-adherent cells.

The obtained primary LA-ADMSC cultures were subcultured up to the 4th passage after reaching ~80% confluency. Cryopreservation at passage 1 and subsequent thawing of cells was performed as previously described [16].

#### 2.1.3. PCS-500-011

Normal human adipose tissue-derived mesenchymal stem cell line PCS-500-011 were purchased from ATTC (ATTC, Manassas, VA, USA). Cells were grown in mesenchymal stem cell basal medium (MSCBM) and supplemented with mesenchymal stem cell growth kit low-serum components (2% FBS, 5 ng/mL rh FGF basic, 5 ng/mL rh FGF acidic, 5 ng/mL rh EGF, 2.4 mM L-Alanyl-L-Glutamine) (ATTC, MA, USA) and 1% Pen/Strep (all Gibco, Manassas, VA, USA). Cells were cultured in a humidified incubator at 37 °C and 5 wt.% CO_2_. Handling, subculturing and maintenance of the cultures were performed according to the manufacturer’s instructions.

### 2.2. Cell Morphology

To assess cell morphology, all three cell types were seeded on 24-well plates in passage 3 and incubated overnight. The next day, cells were fixed and stained for actin filaments (F-actin) using CytoPainter Phalloidin-iFlour 555 reagent (Abcam, Cambridge, UK). Cells were counterstained with DAPI (Sigma-Aldrich, Missouri, MO, USA) and imaged with EVOS FL fluorescence microscope (Thermofisher Scientific, Manassas, VA, USA).

### 2.3. Metabolic Activity

To determine the metabolic activity of the cells, the Alamar Blue assay was performed. Cells were seeded in 48-well plates containing 20,000 cells/well and were allowed to adhere overnight. To examine cell growth/metabolic activity, cells were treated with 10 µg/mL of Alamar Blue (Santa Cruz, Dallas, TX, USA) twice a week for 2 h at 37 °C. The fluorescence intensity of each sample was measured at 530/25 nm excitation and 590/35 nm emission wavelength using a Varioskan Flash multi-well plate reader (Thermo Scientific, Manassas, VA, USA).

### 2.4. RNA Isolation and RT-PCR

RNA was extracted using TRI-reagent (Sigma-Aldrich, St. Louis, MO, USA), as described by the manufacturer, and 1.0 µg of each sample was reverse-transcribed using High Capacity cDNA Reverse Transcription Kit (Applied Biosystems, Thermo Fisher, Waltham, MA, USA). The qRT-PCR was performed on a QuantStudio 12K Flex thermal cycler (Applied Biosystems) with LightCycler 480 SYBR Green I Master (Roche, Basel, Switzerland). Unless otherwise specified, the forward and reverse primers used to detect gene-specific mRNA were designed with Primer3 software, version 4.1.0 (Untergasser et al., Nucleic Acids Res 2012) and produced by Sigma-Aldrich. All the primer pairs are listed in Table 1. The beta-2-microglobulin (*B2M*) was used as the internal control. The mRNA expression data were calculated as log2FC (−ΔΔCt) values. For the purpose of the semi-quantitative evaluation of the qRT-PCR results, the expression levels were considered increased/decreased at log2FC levels >1.00/<−1.00, with partial trends defined at log2FC levels >0.50 and >−0.50.

### 2.5. Cell Characterisation Using Imaging Flow Cytometry 

A characteristic marker expression of cultured ADMSCs was detected by imaging flow cytometric analysis. Briefly, cells were grown for 7–10 days at 37 °C and 5 wt.% CO_2_ and detached from the cell culture plastic at different passages (p2, p3 and p4) with trypsin-EDTA (Thermo Fisher Scientific, MA, USA), fixed with fixative solution (Merck KGaA, Darmstadt, Germany) and labelled with directly labelled antibodies (anti-CD90-APC, anti-CD105-FITC, anti-CD73- PE, anti-CD44-FITC, anti-CD31-AF488, anti-CD34-AF488, anti-CD45-AF488, anti-HLA-DR-PE) for 1 h at RT (all ExBio, Vestec, Czech Republic). Median fluorescence intensity was analysed using multispectral flow cytometry (MIFC) Amnis^®^ ImageStream^®^XMark II (Luminex Corporation, Austin, TX, USA). Cells were gated by forward and side scatter to eliminate debris.

### 2.6. Induction of Cell Differentiation

The trilineage differentiation potential of cultured ADMSCs was induced by incubation in respective differentiation media, followed by differentiation verification, through standard staining methods (Oil Red O, Alizarin Red or Alcian Blue), as described below. Media were changed every 3 to 4 days.

Before differentiation, cells were cultured in mesenchymal stem cell basal medium (ATCC^®^ PCS-500-030™ Mesenchymal Stem Cell Basal Medium for Adipose, Umbilical and Bone Marrow-derived MSCs, ATCC, Manassas, VA, USA) and supplemented with Mesenchymal Stem Cell Growth Kit for Adipose and Umbilical-derived MSCs—Low Serum (ATCC^®^ PCS-500-040™, ATCC, Manassas, VA, USA) and 1 wt.% penicillin/streptomycin (Gibco, Manassas, VA, USA) for at least 48 h. 

To induce differentiation, cells were seeded on 12-well plates at a concentration of 40,000 cells/well and incubated at 37 °C with 5 wt.% CO_2_ for 48 h before differentiation was initiated. For adipogenic differentiation, 1.5 mL of adipocyte differentiation initiation medium (The Adipocyte Differentiation Toolkit, ATCC, Manassas, VA, USA) was added to each well for 48 h to begin the adipocyte differentiation process. After 48 h, the medium was removed, 1.5 mL of Adipocyte Differentiation Maintenance Medium (The Adipocyte Differentiation Toolkit, ATCC, Manassas, VA, USA) was added and adipogenesis was induced for 21 days. Cells were then fixed with a fixative solution (Merck KGaA, Darmstadt, Germany) for 15 min at room temperature (RT) and washed with 60 wt.% isopropanol. Staining with Oil Red O (Sigma-Aldrich, Missouri, MO, USA) for 45 min at RT showed the accumulation of lipid droplets in intracellular vacuoles, indicating adipogenic differentiation. To quantify lipid droplets, cells were first rinsed with PBS and 100% isopropanol was used to elute Oil Red O. After 10 min of incubation, absorbance was measured at 405 nm using a Varioskan Flash multi-well plate reader (Thermo Scientific, Manassas, VA, USA). Osteogenic differentiation was induced in an osteogenic medium (Osteocyte Differentiation Tool, ATCC^®^ PCS-500-052™, Manassas, VA, USA). After 21 days of incubation, the osteogenic phenotype was assessed by staining the cells using Alizarin Red S (ARS, Sigma-Aldrich, Missouri, MO, USA). For quantification of mineralisation, ARS dye was extracted using 10 wt.% acetic acid, neutralised with ammonium hydroxide and absorbance was measured at 405 nm using Varioscan Flash multi-plate reader (Thermo Scientific, Manassas, VA, USA).

Chondrogenic differentiation of ADMSC was induced in the chondrogenic medium (Chondrocyte Differentiation Tool, ATCC^®^ PCS-500-051™, Manassas, VA, USA). After 21 days, the chondrogenic phenotype was assessed by Alcian Blue staining (Merck, Darmstadt, Germany).

### 2.7. Cell Encapsulation and Staining

According to the Meufacuturer’s Guide (Chondrocyte Differentiation Tool ATCC PCS -500-051), chondrocyte differentiation requires that cells are to be grown in a three-dimensional aggregate cell culture to provide a scaffold for the deposition of proteoglycans. For this purpose, 0.025 g of sodium alginate was dissolved in 2 mL of ultrapure water (1.25 wt.%) and filtered using a sterile syringe filter with 0.22 µm pore size. A total of 80 µL of the filtered alginate solution was added to 20 µL of cell suspension (with a concentration of 3 million cells per ml) in the culture medium to reach a final concentration of 1 wt.% alginate and 600,000 cells per ml.

The bioink was dispensed in 2 µL aliquots into a Petri dish filled with sterile 0.2 wt.% CaCl_2_ solution using a digital pipette. After all of the capsules were produced, the 0.2 wt.% CaCl_2_ solution was replaced with sterile 5 wt.% CaCl_2_ solution for 1 min. Afterwards, the CaCl_2_ solution was removed and the capsules were resuspended with the culture medium 3 times for 5 min. Finally, the capsules were transferred to a differentiation medium and incubated at 37 °C and 5% CO_2_. After 21 days of differentiation, encapsulated cells were fixed with a fixative solution (Merck KGaA, Darmstadt, Germany) and stained for aggrecan using Ms mAb anti-AGR (Abcam, Cambridge, UK) and collagen type II using Rb pAb anti-COLIIA1 (Abcam, Cambridge, UK). The secondary antibodies Goat pAb to Rb—AF 594 (Abcam, Cambridge, UK) and Rb pAb to Ms—AF 488 (Abcam, Cambridge, UK) were used. The capsules were visualised using a fluorescent microscope EVOS FL (Thermo Fisher Scientific, Manassas, VA, USA).

### 2.8. Statistical Analysis

All numerical values are expressed as mean ± standard deviation (SD). The Shapiro–Wilk test confirmed the normal distribution of the experimental data. Levene’s test was used to assess the equality of variances. Since all data sets were well modelled by a normal distribution and homoscedastic, a one-way analysis of variance (ANOVA) followed by Bonferroni post hoc test were performed. Determined *p*-values of <0.05 were considered statistically significant. Statistical analysis was performed using SPPS Statistics 27 (IBM Corp. Armonk, NY, USA).

## 3. Results

### 3.1. Isolation of ADMSCs

PV-ADMSCs were isolated from the paravertebral adipose tissue of a 3-month-old child who underwent surgery for tethered cord syndrome. This is a clinical condition that can occur in children and adults. It has many causes, leading to tension in the spinal cord, resulting in neurologic and orthopaedic symptoms. Typical radiographic findings may include a deep location of the conus medullaris with fatty infiltration of the *filum terminale* [14,15]. Other types of spinal dysgraphia may also be present, such as meningocele, myelomeningocele, lipomyelomeningocele, cleft spinal cord malformations, dermal sinus tracts, anorectal malformations and various intraspinal tumours. Treatment is surgical with correction and reconstruction of the underlying pathology [20].

LA-ADMSCs were isolated from lipoaspirates of five adult female donors (mean age 48.4 years, Supplementary Table S1). Compared to the primary lipoaspirates, the first passages of the isolated primary cultures expressed markedly more mesenchymal stem markers, *CD44*, *ENG*, *THY1*, *NT5E* (log2FC: 2.29 ± 0.54, 3.12 ± 0.54, 2.25 ± 0.63 and 2.82 ± 0.44, respectively) and fewer negative markers *CD34* (a haematopoietic stem cell marker, log2FC: −2.29 ± 1.22), *PECAM1* (a vascular endothelial marker, log2FC: −0.98 ± 1.85), *PTPRC*, *HLA-DRA* (haematopoietic markers; log2FC −1.98 ± 0.89 and −3.72 ± 2.22, respectively), confirming that the cultured cells are indeed mesenchymal stem cells (Supplementary Figure S2a). Looking at the individual cultures, the LA-ADMSCs from donor 4 expressed the highest levels of *CD44*, *ENG* and *NT5E*, second highest level of *THY1*, the lowest levels of *PECAM1*, *PTPRC* and *HLA-DRA* and second lowest level of *CD34* (Supplementary Figure S2b,c). For this reason, the LA-ADMSCs from donor 4 were used in all of the subsequent comparisons and experiments.

PCS-500-011 is a commercial normal human adipose-derived mesenchymal stem cell line from an adult donor. The cells were cryopreserved in the second passage to ensure the highest viability and plating efficiency. Specific staining revealed that the cells were positive for CD29, CD44, CD73, CD90, CD105 and CD166 and negative for CD14, CD31, CD34 and CD45 (adipose-derived mesenchymal stem cells; normal, human (ATCC PCS-500-011)). The cell line has been used to date for various research applications, including adipose tissue regeneration [21], osteointegration in bioactive coatings [22], chondrogenesis [23] and neurogenesis [24].

### 3.2. Morphology and Growth Characteristics

**Figure 1:**
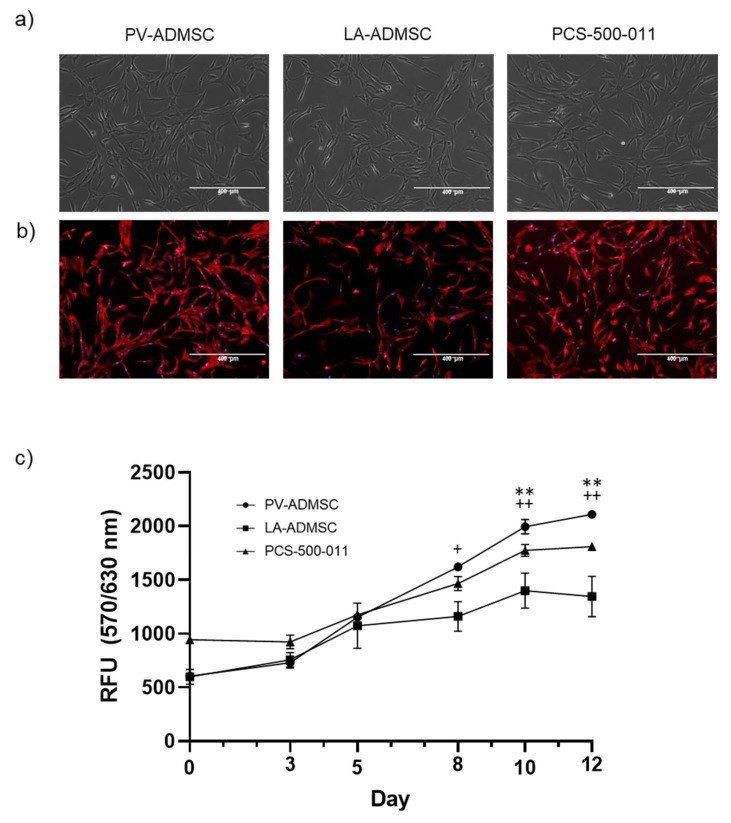
Morphology and growth characteristics of ADMSCs obtained from different sources. (**a**) Characteristic phase contrast microscopy in passage 3 (bar: 400 µm); (**b**) fluorescent staining of actin filaments in passage 3, nuclei were counterstained with DAPI (bar: 400 µm); (**c**) metabolic activity assessment using Alamar Blue (resazurin) assay. Differences between PV and PCS: ** *p* < 0.01; differences between PV and LA: + *p* < 0.05, ++ *p* < 0.01 were calculated using ANOVA.

An early passage (p1–p3) of all three cell types was cultured in MSCBM and supplemented with a mesenchymal stem cell growth kit. The cells showed plastic adherence and a spindle-shaped fibroblastoid morphology (Figure 1a). Cell morphology was also characterised with a fluorescent phalloidin conjugate, staining actin filaments (red) and DAPI nuclear staining (blue) (Figure 1b). No significant morphological differences were observed between the three used ADMSCs. 

We used the Alamar Blue assay to analyse cell growth as a direct indicator of metabolically active healthy cells. Measurements over 12 days showed a significant increase in metabolism, indicating continued cell growth in all cell types (Figure 1c). When considering the differences between cell types, we noted increased cell growth of PV-AMDSC at 8 days compared to LA-ADMSC. Furthermore, on days 10 and 12, LA-ADMSC and PCS cells grew significantly slower than PV-ADMSC, likely due to contact inhibition.

### 3.3. Stem Marker Gene Expression

**Figure 2:**
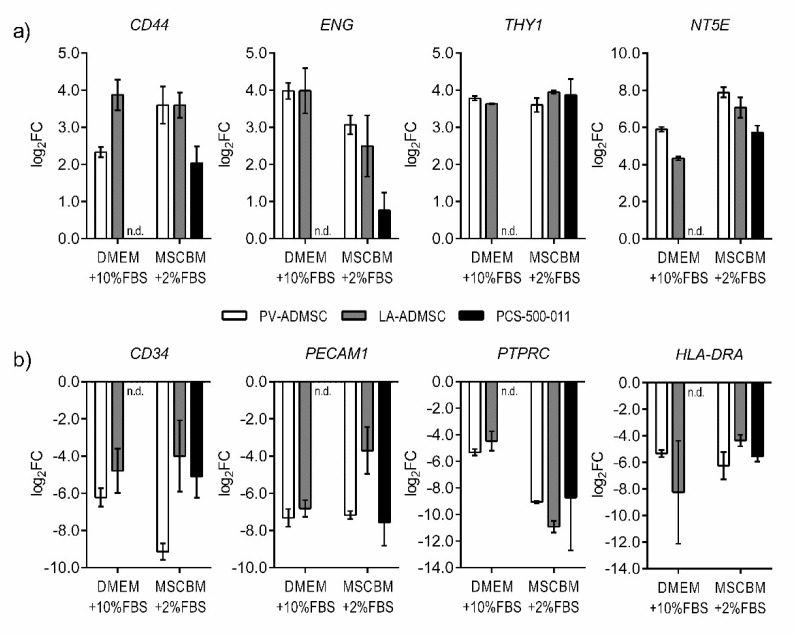
Gene expression in human ADMSCs of different origins. The cells were cultured in 10% FBS supplemented DMEM or 2% FBS supplemented MSCBM media. The results from the 2nd and 3rd passages were pooled together (*n* = 2) and normalised to the original lipoaspirate from donor 4. (**a**) Gene expression of the mesenchymal stem markers *CD44*, *ENG*, *THY1* and *NT5E*. (**b**) Gene expression of the negative markers, *CD34*, *PECAM1*, *PTPRC* and *HLA-DRA*. (n.d.—no data).

We cultured the PV-ADMSC and LA-ADMSC cells for three passages in DMEM enriched with 10 wt.% FBS and measured the gene expression of MSCs and negative markers in the second and third passages (Figure 2a,b). The PV-ADMSCs expressed more *NT5E* (log2 FC 1.57) but less *CD44* (log2FC −1.54) than the LA-ADMSCs. No significant difference was observed in the expression of *ENG* or *THY1*. As for negative markers, the PV-ADMSCs expressed fewer *CD34* (log2FC −1.44) with a trend towards lower *PECAM1* and *PTPRC* levels (log2FC −0.50 and −0.85, respectively), but more *HLA-DRA* (log2FC 2.92) than the LA-ADMSCs.

To allow direct comparison with the commercially available ADMSCs, the PCS-500-011 cells, both cell types, were also cultured in parallel in the 2 wt.% FBS supplemented MSCBM medium recommended for the PCS-500-011 cells (see Materials and Methods). For the most part, culturing in MSCBM media had a positive effect on the PV-ADMSCs, increasing the expression of *CD44* and *NT5E* stem markers (Figure 2a, log2FC 1.26 and 1.99, respectively), while decreasing the expression of the negative markers *CD34*, *PTPRC* and, to some extent, *HLA-DRA* (Figure 2b, log2FC −2.92, −3.75 and −0.92, respectively). The expression of *ENG* (log2FC −0.92) also decreased somewhat, while no major changes were observed in the expression of *THY1* or *PECAM1*. *MSCBM* medium also increased the *NT5E* expression in LA-ADMSC cells (Figure 2a, log2FC 2.74). Still, it negatively affected the expression of *ENG* (log2FC −1.49) and increased the expression of the negative markers CD34, PECAM1 and HLA-DRA (Figure 2b, log2FC 0.79 3.12 and 3.89, respectively). The expression of *PTPRC* was strongly decreased (log2FC −6.46), but no significant effect was observed for the expression of *CD44* and *THY1*.

When comparing the two cell types cultured in MSCBM, the differences in MSC markers were smaller, with a trend towards higher *ENG* and *NT5E* in PV-ADMSCs (Figure 2a, log2FC 0.57 and 0.81, respectively). For negative markers, the differences between the two cell types were greater than in DMEM media, with PV-ADMSCs expressing lower levels of *CD34, PECAM1* and *HLA-DRA* (Figure 2b, log2FC −5.15, −3.47, and −1.90, respectively), but higher levels of *PTPRC* (log2FC 1.86) than the LA ADMSCs.

Compared to the commercially available ADMSCs (PCS-500-011), both the PV-ADMSCs and the LA-ADMSCs expressed more *CD44* (log2FC 1.56 for both), *ENG* (log2FC 2.30 and 1.73, respectively) and *NT5E* (log2FC 2.16 and 1.35, respectively), while there is not much difference in *THY1* levels (Figure 2a). For the negative markers, the picture is not quite as clear (Figure 2b). The PV-ADMSCs express less *CD34* and, to some extent, less *HLA-DRA* than PCS-500-011 (log2FC −4.05 and −0.71, respectively); LA-ADMSCs express less *PTPRC* but more *CD34, PECAM1* and *HLA-DRA* than PCS-500-011 (log2FC −2.19, 1.10, 3.86 and 1.19, respectively).

Overall, the mRNA results (qRT-CPR) show that using a special stem cell medium (MSCBM with 2 wt.% FBS) instead of a standard medium with 10 wt.% FBS is beneficial for culturing PV-ADMSCs, but not so much for culturing LA-ADMSCs. Comparing the three types of ADMSCs, PV-ADMSCs seem to express, on average, the highest levels of MSC markers and the lowest levels of negative markers.

### 3.4. Stem Marker Protein Expression

Cells cultured in MSCBM medium containing 2 wt.% FBS were analysed for protein expression of MSC, and negative markers in the second, third, and fourth passages by multispectral imaging flow cytometry. Area and aspect ratio of brightfield images were used to identify the cell population of interest and exclude doublets and debris. Gradient RSM was used to draw additional gates to exclude blurred cell images, followed by gating for fluorescence positives using the fluorescence intensity histograms of channels 2, 3 and 9. 

**Figure 3:**
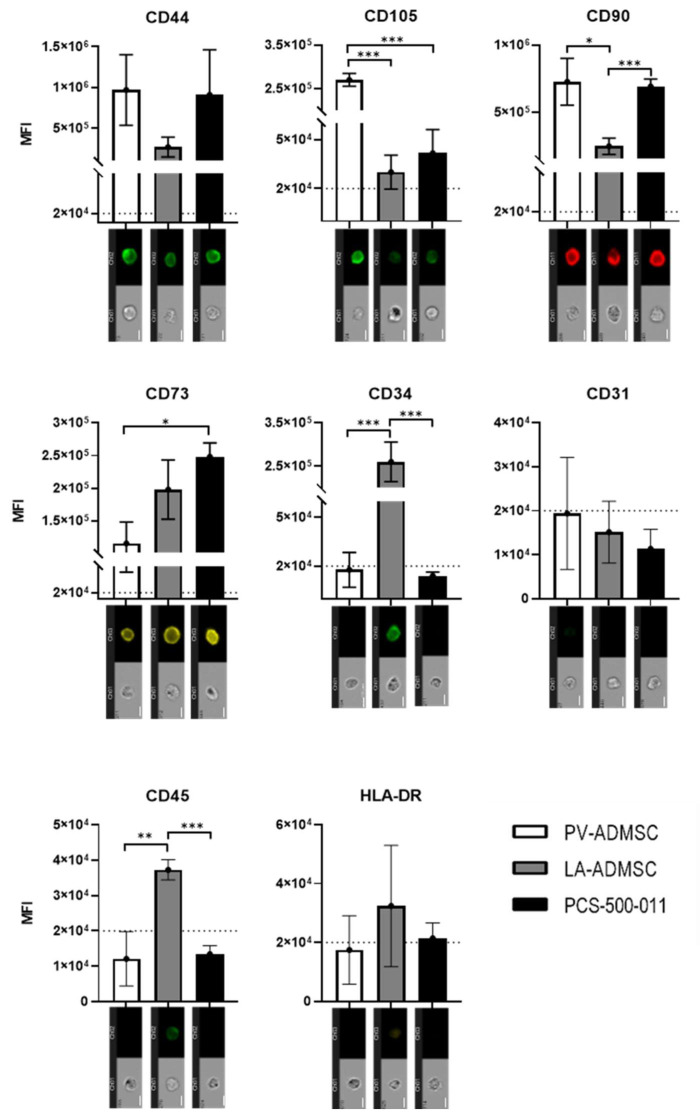
MSC protein marker analysis by imaging flow cytometry. Cells were cultured in MSCBM, harvested at passages 2, 3 and 4, and fixed. Monoclonal antibodies conjugated to FITC (CD44, CD105), AF488 (CD31, CD34, CD45), PE (CD73, HLA-DR) and APC (CD90) were used to stain the cells. One thousand events were analysed for cell type and protein markers. Values are expressed as median fluorescence intensity (MFI), with error bars representing the mean ± S.D. of three independent samples (p2–p4). Differences between the cells: * *p* < 0.05, ** *p* < 0.01, *** *p* < 0.001 were calculated using ANOVA. Representative images are shown below each bar (scale = 10 µm). The dashed line represents a threshold of 2 × 10^4^ MFI above which the signal was considered positive.

Protein analysis of PV-ADMSC, LA-ADMSC and PCS-500-011 confirmed that all three cell types expressed CD44, CD105, CD90 and CD73 (Figure 3). Overall, LA-ADMSC appeared to have the lowest expression of MSC markers. In contrast, the fluorescence intensity of CD105 was up to 5-fold higher in PV-ADMSC than in the other two cell types, whose measured fluorescence was just above the threshold. The commercial cell line PCS-500-011 had the highest expression of CD73, while no significant differences were found for CD44 between all tested cell types.

Regarding the negative markers, LA-ADMSC had the highest values for negative markers compared to PV-AMDSC and PCS-500-001. While no significant differences were found for CD31 and HLA-DR, the expression of CD45 was above the threshold and significantly higher in LA-ADMSC. For CD34, whose role as a negative MSC marker in the stem cell community is still contradictory [25], a strong expression was observed in LA-ADMSC, while no expression was found in PV-ADMSC and PCS-500-011. Overall, the trend suggests that LA-ADMSCs express the lowest amount of positive MSC markers and the highest amount of negative MSC markers. 

### 3.5. Adipogenic Differentiation of MSCs

**Figure 4:**
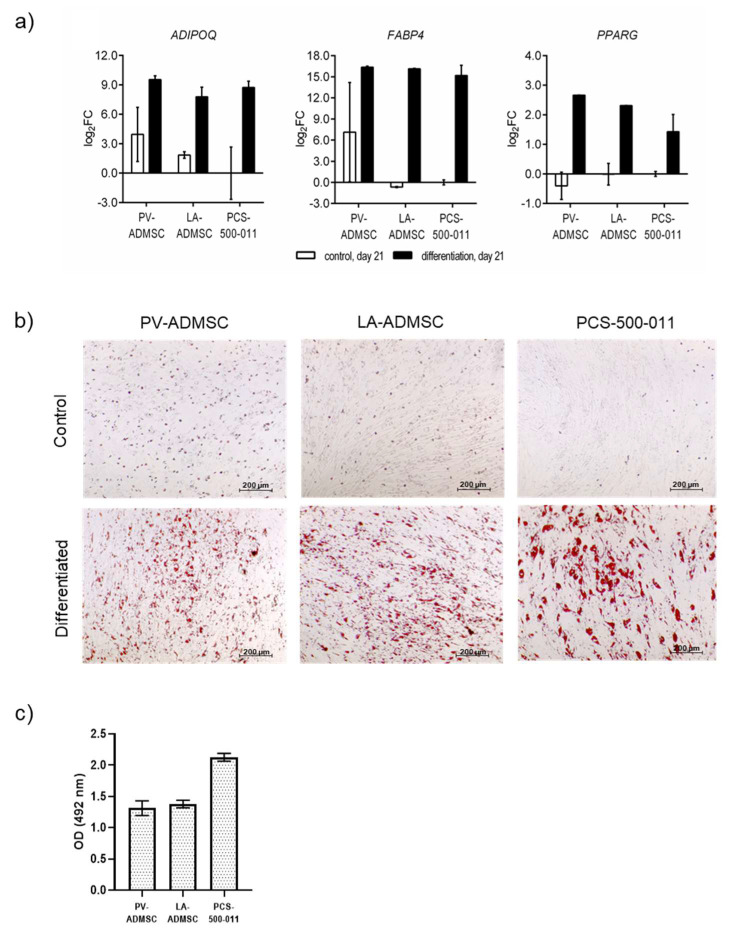
Adipogenic differentiation of human ADMSCs. Cells were cultured in adipogenic medium (differentiated) and standard AMDSC growth medium (control). (**a**) The mRNA expression of *ADIPOQ*, *FABP4* and *PPARG* in human ADMSCs after 21 days of differentiation. The results are normalized to the control (undifferentiated) PCS-500-011 cells on day 21 (*n* = 2). (**b**) Microscopic image of ADMSCs culture in standard ADMSC growth medium (upper panel) or adipogenic medium (lower panel). After 21 days of incubation, cells were fixed and stained with Oil Red O for natural lipids. (**c**) Lipid droplets were quantified by extracting the stain with isopropanol, followed by absorbance measurement at 492 nm. Data are expressed as mean (*n* = 2), with error bars representing standard deviation.

The cells were further characterised regarding their ability for tri-linear differentiation. First, we looked at the adipogenic potential. After 21 days of differentiation, gene expression of the adipogenic markers, *ADIPOQ*, *FABP4* (involved in adipose and fatty acid metabolism, respectively) and *PPARγ* (regulator of adipogenic differentiation) were measured (Figure 4a). In all three cell types, all three adipogenic markers were markedly enhanced—*ADIPOQ* by 8.74–9.54-fold, *FABP4* by 15.19–16.36-fold and *PPARG* by 1.43–2.66-fold (log2FC in all cases)—compared to the undifferentiated control cells, respectively. The 21-day differentiated PV-ADMSCs showed the highest expression of all three adipogenic markers compared to the differentiated LA-ADMSCs and PCS-500-011 cells (log2FC 0.22–1.75). On the other hand, differentiated PCS-500-011 showed the lowest levels of adipogenic markers, *FABP4* and *PPARG*.

In the same experimental setup after 21 days of incubation, we examined the accumulation of lipid droplets using Oil Red O staining. We confirmed that all three cell types accumulated lipid droplets, as shown in Figure 4b (bottom panel). Interestingly, the strongest accumulation of lipid droplets was observed in the PCS-500-011 cell line, which was also confirmed by the extraction and quantification of lipid droplets using spectrophotometry (Figure 4c). 

### 3.6. Osteogenic Differentiation of MSCs 

**Figure 5:**
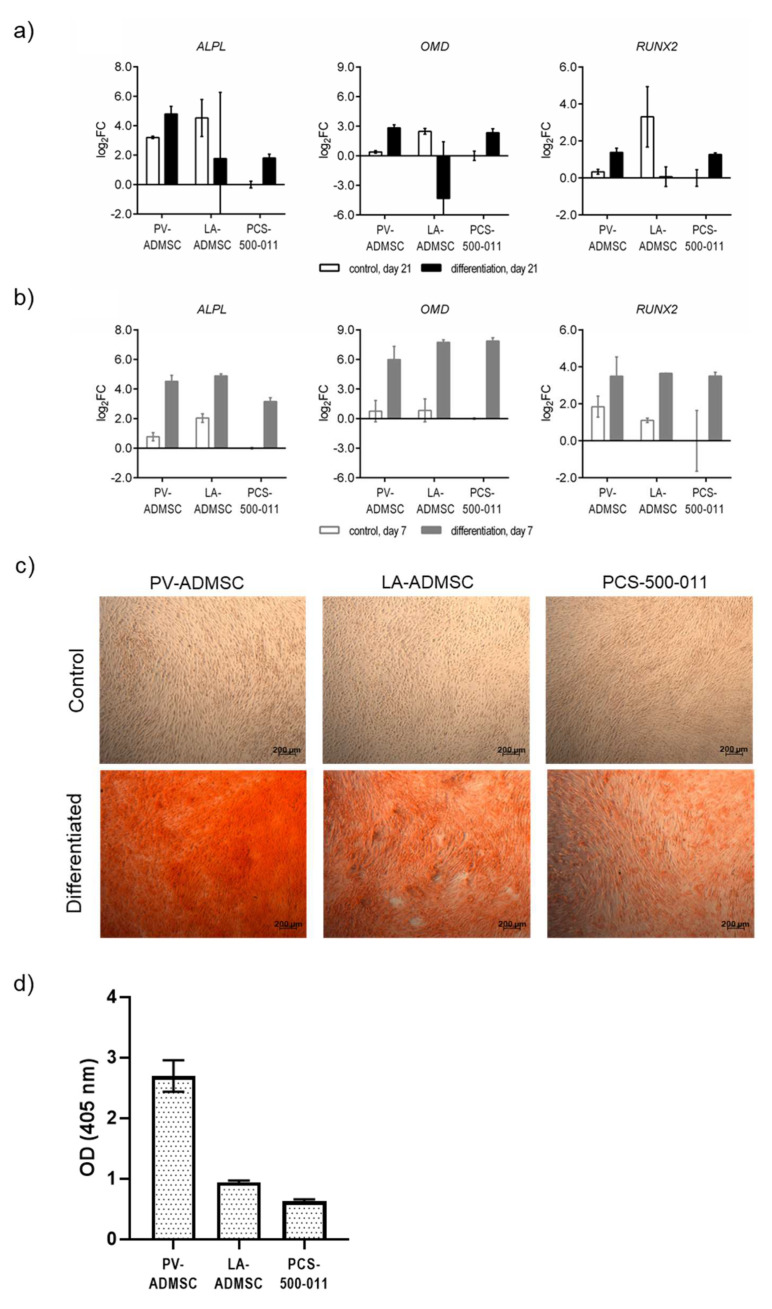
Osteogenic differentiation of human ADMSCs. (**a**) The mRNA expression of *ALPL*, *OMD* and *RUNX2* in human ADMSCs after 21 (days) and (**b**) 7 days of differentiation. The results are normalized to the control (undifferentiated) PCS-500-011 cells on day 7 and 21, respectively (*n* = 2). (**c**) After 21 days of incubation in either standard or differentiation medium, cultures were fixed and stained with Alizarine Res S (ARS). Calcium deposits are stained in red. (**d**) Quantification of ARS was performed by extracting the mineral deposits with acetic acid and measuring the absorbance at 405 nm. Data are given as mean (*n* = 2), with error bars representing standard deviation.

We next examined the osteogenic potential of the three types of ADMSCs. At the mRNA level, both 21-day differentiated PV-ADMSCs and PCS-500-011 cells expressed increased levels of all three osteogenic markers, *ALPL* (log2FC 4.79 and 1.81, respectively), *OMD* (log2FC 2.83 and 2.35, respectively), and *RUNX2* (log2FC 1.38 and 1.27, respectively), compared with nondifferentiated control cells (Figure 5a). PV-ADMSCs expressed the highest levels for all three genes. On the other hand, differentiated LA-ADMSCs expressed the lowest levels of the three osteogenic markers, with only *ALPL* levels increasing above the control level (log2FC 1.77); there was no difference for *RUNX2*, while *OMD* levels actually fell well below the control level (log2FC −4.33). Since it has been previously described that the expression of certain osteogenic markers occurs earlier in the differentiation process [26], we also checked the mRNA levels of the three osteogenic markers after only 7 days of differentiation (Figure 5b). At this time point, all three cell types showed increased expression of all three osteogenic markers compared to the control and the undifferentiated cells (log2FC 3.15–4.88 for *ALPL*, 5.99–7.87 for *OMD*, and 3.50 to 3.65 for *RUNX2*). Moreover, these expression levels were higher than at 21 days in all but one case (*ALPL* in PV-ADMSCs). In contrast to 21 days, after 7 days of differentiation, LA-ADMSCs showed the highest levels of *ALPL* and *RUNX2*, and PV-ADMSCs showed the lowest levels of *OMD* and RUNX2.

Phenotypic analysis by Alizarine Red staining (ARS) after 21 days confirmed the presence of mineral deposits (bright red) in all three cell types compared with the control (Figure 5c). It was evident that PV-ADMSC showed the strongest mineralisation, which was further investigated by ARS extraction with acetic acid. The spectrophotometric analysis confirmed that PV-ADMSC produced three times more calcium deposits than the other two cell types (Figure 5d). 

### 3.7. Chondrogenic Differentiation of MSCs

**Figure 6:**
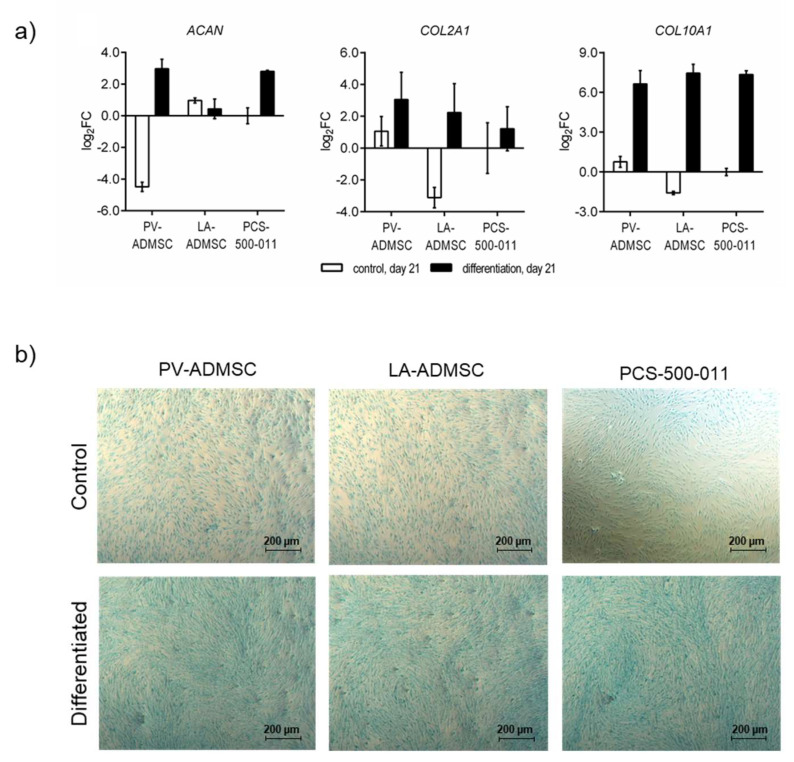
Chondrogenic differentiation of human ADMSCs. (**a**) The mRNA expression of *ACAN*, *COL2A1* and *COL10A1* in human ADMSCs after 21 days of differentiation. The results are normalized to the control (undifferentiated) PCS-500-011 cells on day 21 (*n* = 2). (**b**) Furthermore, after 21 days of incubation in either standard (upper panel) or differentiation medium (lower panel), cells were fixed and stained with Alcian Blue staining solution for glycosaminoglycans.

Last, we also determined the chondrogenic potential of the three ADMSC sources. After 21 days of chondrogenic differentiation, all three cell types showed increased mRNA expression of *COL2A1* (log2FC 1.22–3.06), but even more of *COL10A1* (log2FC 6.65–7.45), a marker for hypertrophic chondrocytes involved in endochondral ossification, which contributes to longitudinal growth and is gradually replaced by bone [27]. PV-ADMSCs and PCS-500-011 cells, but not LA-ADMSCs, also expressed increased *ACAN* compared to undifferentiated controls (log2FC 2.97 and 2.80, respectively). Comparing the three cell types, the differentiated PV-ADMSCs expressed the highest levels of both chondrogenic markers, *ACAN* and *COL2A1,* and the lowest levels of the hypertrophic marker, *COL10A1* (Figure 6a).

After 21 days of incubation, the cells grown in the chondrocyte differentiation medium showed an intense blue colour when stained with Alcian Blue, indicating the presence of proteoglycans in the extracellular matrix. In contrast, cells grown in the normal growth medium show a light blue colour (Figure 6b).

**Figure 7:**
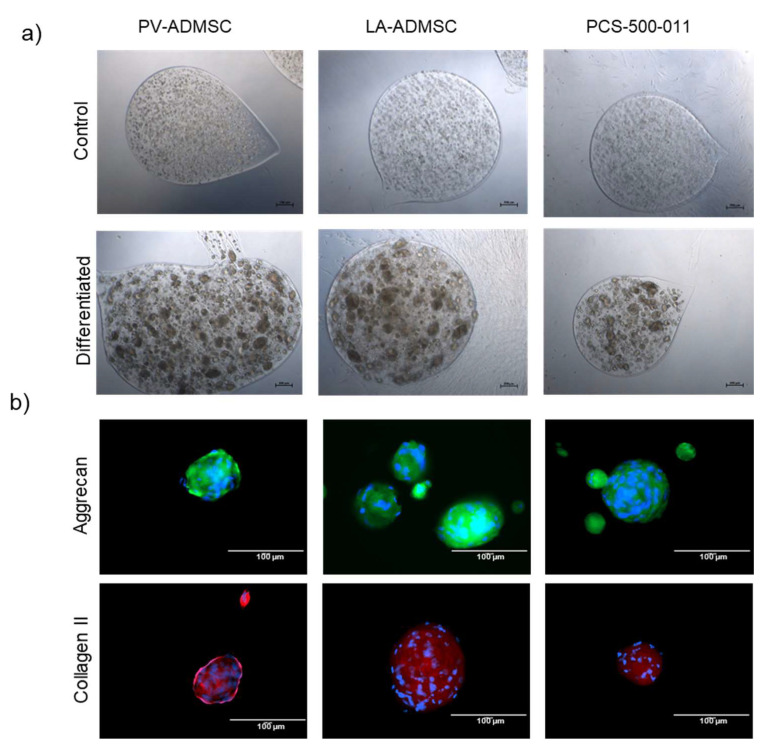
Chondrogenic differentiation in alginate encapsulated human LA-ADMSCs. (**a**) Bright-field microscopic pictures of cells encapsulated in alginate. The upper panel shows cells grown in MSCBM and the lower panel in the chondrocyte differentiation medium. (**b**) Immuno-stained differentiated cell aggregates were stained for aggrecan (upper panel) and type II collagen (lower panel) and assessed by fluorescent microscopy.

While phenotypic analysis with Alcian Blue confirmed, to some extent, the presence of a proteoglycan-containing extracellular matrix in 2D cultures, standard chondrogenic differentiation of MSCs usually requires a 3D cell environment [28]. For this purpose, we developed alginate microcapsules, as recommended by the ATCC, to provide a scaffold for the deposition of ECM components. After 21 days in the chondrocyte differentiation medium, we observed dark cell aggregates of 50–100 µm in size for all cell types, whereas no aggregates were seen in the control samples (Figure 7a). When staining the cells for aggrecan and type II collagen, the control alginate microcapsules, but not the aggregated cells, fell apart, allowing us to confirm the presence of both extracellular matrix proteins for all three cell types (Figure 7b).

## 4. Discussion

Adipose tissue-derived MSCs are already a widely used cell type in research and clinical applications, and the range of potential specific applications is still growing. Due to their ease of collection, abundance in the adult population, high yield and potential to differentiate into three developmental lineages, ADMSCs have good potential for a wide range of applications in regenerative medicine. At the time of this study, there were approximately 350 clinical trials listed on ClinicalTrials.gov covering a wide range of applications, including soft tissue regeneration, skeletal tissue repair, ischemic injury, immune disorders and myocardial infarction, to name a few [29]. Although some promising results have already been published, the translation into regular clinical practice has not yet seen its full potential. 

It is becoming increasingly clear that the origin of stem cells plays a crucial role in determining their behaviour and, thus, their potential therapeutic applications. With this in mind, it is important to search for new, easily accessible tissue sources of ADMSCs, apart from the traditional sources (e.g., lipoaspirates). Herein, we aimed to explore the potential of a new source of ADMSCs (termed PV-ADMSCs) from a paediatric patient, obtained from paravertebral adipose tissue during routine spinal cord surgery, without risk to the patient. To increase the translational potential of this new source, we also used and optimised a relatively simple isolation procedure. To understand the capacity of this new ADMSC source, we compared its properties with ADMSCs from two other sources (i.e., AD-MSC, after “traditional” isolation from lipoaspirate and commercially available ADMSCs from ATCC). 

Our data show that PV-ADMSCs have a stronger proliferative capacity than the two sources tested. This is consistent with previous studies investigating the in vitro replication of paediatric MSCs compared to adult MSCs [30,31], and may be of particular interest for applications requiring rapid expansion and a large cell population. This increased regenerative capacity and ease of isolation could open new avenues for the clinical use of PV-AMDSCs, or at least expand the potential range of possible sources for paediatric ADMSCs.

This study confirmed successful trilineage differentiation of the isolated PV-ADMSCs, confirming their stem cell property and their comparability with ADMSCs from other sources. Furthermore, we found significant differences in the expression of positive and negative stem cell markers between the compared cell types. Overall, PV-ADMSCs showed increased expression of stem cell markers, such as CD105, which were previously associated with increased cell proliferation and colony formation in ADMSCs [32]. In addition, it has been reported that CD105-positive cells within the population of BMSCs exhibit greater stemness [33] and increased chondrogenic potential [34]. Although we could not quantify the chondrogenic potential in our tested cell types due to the different size of the cell aggregates, we still observed strong chondrogenic differentiation in PV-ADMSCs in 2D and 3D. Nevertheless, we demonstrate the importance of using 3D models to successfully assess chondrogenic differentiation and provide a potential future model that could be used for this purpose. However, standardisation in this regard would be necessary to obtain a comparable analysis between laboratories. 

Our data also show that PV-ADMSCs express ~3-fold (log2FC) more *ALPL* at day 21, while the other two cell types have similar levels of ALPL, a gene involved in bone mineralisation [35]. We also confirmed strong osteogenic differentiation of PV-AMDSCs by Alizarine Red staining (used as a measure of mineralisation). These data could be a good indicator for further use in osteochondral applications.

Our results suggest that paediatric paravertebral adipose depots contain a pool of stem cells with the capacity for self-renewal, multipotency and the ability to differentiate into the functional mature phenotype of adipogenic, osteogenic and chondrogenic lineage. To our knowledge, this is the first case of stem cells isolated from paravertebral tissue in a paediatric patient. We demonstrated that PV-ADMSCs have a higher proliferative potential and strong chondrogenic and osteogenic differentiation potential than ADMSCs from other sources. As such, PV-ADMSCs could have prominent clinical implications in skeletal tissue repair and regeneration, including bone fractures, craniofacial defects, vertebral disk regeneration, repair of tendon and ligaments, osteoarthritis and osteoarticular repair [36,37,38]. 

Therefore, we believe that PV-ADMSCs are a promising cell source for further studies on the exploration and therapeutic application of MSCs. Considering the simplicity of the isolation protocol used, the growth characteristics of the newly isolated PV-ADMSCs and the preservation of the most important markers up to the 5th passage, we believe that this approach is easily transferable to other laboratories with basic cell biology equipment and access to clinical samples.

## 5. Conclusions

In this study, we defined and confirmed a new source of ADMSCs, obtained from a “waste” biological sample from a regular surgery in a paediatric patient. To date, this is the first such isolation and presents a potentially crucial future source for use in regenerative medicine. Considering its origin and other defined properties, several conclusions can be made. In summary:

- The regenerative power of the paediatric patient as the source of the ADMSCs cannot be overlooked and present an important advantage in potential clinical applications.
- Since the source sample was obtained in regular surgery, such procedures could be a yet unexploited future source of stem cells and lead to crucial new cell therapies in the future.
- Since our isolation relies on a waste product during surgery, it also does not pose any additional harm to the patient and is ethically acceptable.
- Using available separation technologies (e.g., MACS), this ADMSC source could be even directly applied after harvesting in patients, suitable for such cell therapy.

## Figures and Tables

**Table 1:** qRT-PCR primers.

Gene Name	Gene ID	Function	Forward Primer (5′→3′)	Reverse Primer (5′→3′)
*CD44*	960	Mesenchymal stem cell markers	GAAGAAGGTGTGGGCAGAAG	TCTGCAGGTTCCTTGTCTCA
*ENG*	2022		GAGGCGGTGGTCAATATCCT	GGACACTCTGACCTGCACAA
			
*NT5E*	4907		TCTCTCAAATCCAGGGACAAATT	GTCCACACCCCTCACTTTCT
*THY*1	7070		CTAACAGTCTTGCAGGTCTCC	CTTCTTTGTCTCACGGGTCAG
*CD34*	947	Haematopoietic stem cell marker	TCTTGGCCAACAGAACAGAAAT	ATAGCCAGTGATGCCCAAGA
*PECAM1*	5175	Vascular endothelial marker	TGACCCTTCTGCTCTGTTCA	CTGAGGCTTGACGTGAGAGG
*HLA-DRA*	3122	Haematopoietic markers	CTCAAGCACTGGGAGTTTGA	CGTTCTGCTGCATTGCTTT
*PTPRC*	5788		ACTACTCCATCTAAGCCAACATG	CACCTCATTGTTTGTGCAAGT
*ADIPOQ*	9370	Adipogenic markers	TGACATCAGGGCTCAGGAT	GGTGCCATCTCTGCCATCAC
*FABP4*	2167		GCCAGGAATTTGACGAAGTCAC	TTCTGCACATGTACCAGGACAC
*PPARG*	5468		GACCACTCCCACTCCTTTGA	TCCACTTTGATTGCACTTTGGTA
*ALPL* [17]	249	Osteogenic markers	GACCTCCTCGGAAGACACTC	TGAAGGGCTTCTTGTCTGTG
*OMD*	4958		ACCCGAGTGTTTTCCAAGAAG	TGGTCATAGTCTTCATCCCACT
*RUNX2*	860		CAACTTCCTGTGCTCGGTG	CTCTTGCCTCGTCCACTCC
*ACAN* [18]	176	Chondrogenic markers	TGAGGAGGGCTGGAACAAGTACC	GGAGGTGGTAATTGCAGGGAACA
*COL2A1* [18]	1280		TTTCCCAGGTCAAGATGGTC	CTGCAGCACCTGTCTCACCA
*COL10A1* [19]	1300		GGCAACAGCATTATGACC	GATGATGGCACTCCCTGAA
*B2M*	567	Internal control	TTCTGGCCTGGAGGCTATC	TCAGGAAATTTGACTTTCCATTC

## Supplementary Materials

The following are available online at https://www.mdpi.com/article/10.3390/biomedicines10020378/s1, Figure S1: Expression of mesenchymal stem and negative markers in LA-ADMSC primary cultures. Table S1: Donor data. Figure S2: Expression of mesenchymal stem and negative markers in LA-ADMSC primary cultures.
