# Supplementary material for: Mesenchymal Stem Cells Isolated from Paediatric Paravertebral Adipose Tissue Show Strong Osteogenic Potential
Source: Biomedicines. 2022 Feb 4;10(2):378. doi: 10.3390/biomedicines10020378 (PMC8962402; doi:10.3390/biomedicines10020378)
Supplement: Supplementary file 1 [file biomedicines-10-00378-s001.zip › biomedicines-1525886-supplementary.pdf]

biomedicines

MDPI

Supplementary Material

Table S1. Donor data.

Cells	Donor:	Sex	Age	Diagnosis	Operation
LA180905	Donor 1	Female	66	Breast cancer	Bilateral breast reconstruction with abdominal-free flaps
LA181015	Donor 2	Female	35	Excess skin after morbid obesity and radical weight loss	Lower body lift
LA181107	Donor 3	Female	42	Breast cancer	Breast reconstruction with abdominal-free flaps
LA190318	Donor 4	Female	56	Breast cancer	Breast reconstruction with abdominal-free flaps
LA190826	Donor 5	Female	43	Breast cancer	Bilateral breast reconstruction with abdominal-free flaps

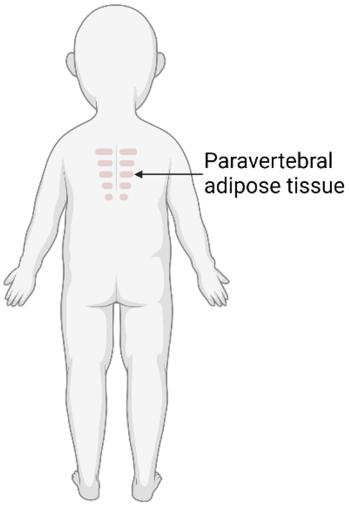

Figure S1. Site of paravertebral adipose tissue deposits in infants.

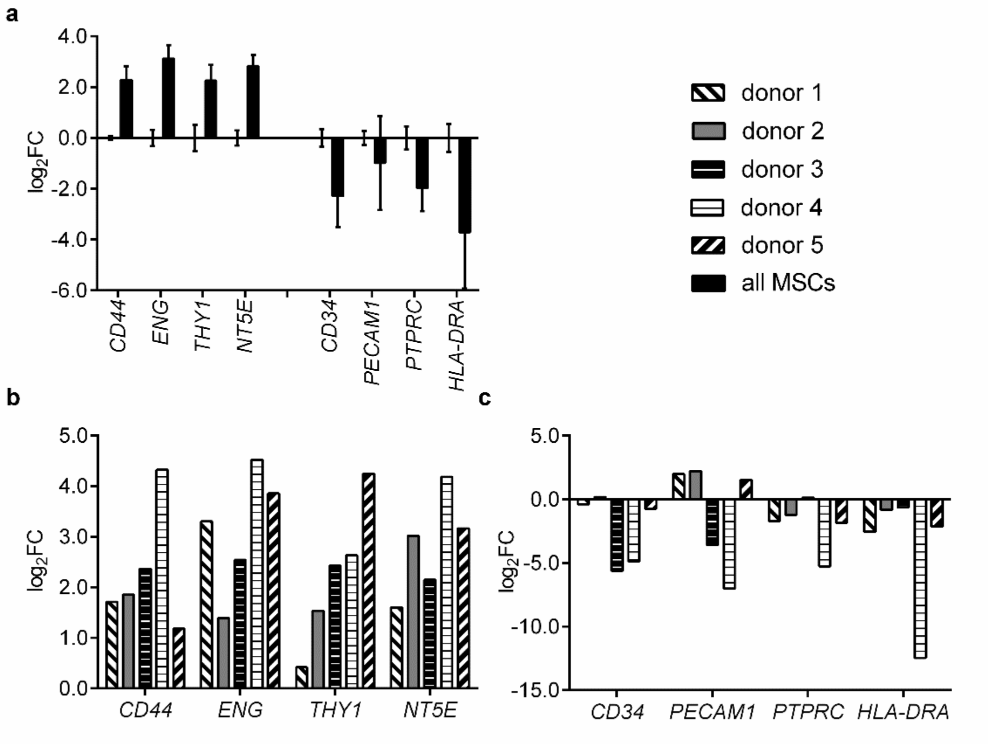

Figure S2. Expression of mesenchymal stem and negative markers in LA-ADMSC primary cultures. (a) Mean mRNA expression of mesenchymal stem cell markers, *CD44*, *ENG*, *THY1*, *NT5E* and of negative markers, *CD34*, *PECAM1*, *PTPRC*, *HLA-DRA* in the first passages of the LA-ADMSC cultures. The results are normalised to the primary lipoaspirates obtained from donors (n = 5). (b) The mRNA expression of the mesenchymal stem cell markers in the individual LA-ADMSC primary cultures isolated from donors 1-5. (c) The mRNA expression of the negative markers in the individual LA-ADMSC primary cultures isolated from donors 1-5.
